# Lead in the Treatment of Perforated Roots*Read before the American Dental Society of Europe, April, 1912.

**Published:** 1912-10-15

**Authors:** J. H. Spaulding

**Affiliations:** Paris, France


### LEAD IN THE TREATMENT OF PERFORATED ROOTS.*

BY DR. J. H. SPAULDING, PARIS, FRANCE.

In presenting for your consideration my experience in the use of lead in the treatment of perforations and certain cases of devitalized teeth, I feel that I am communicating a method of treatment which, though not new, is at least not commonly practiced, but yet which, in my estimation, is of the highest value, for in all the domain of practical dentistry there is, perhaps, no more difficult and annoying

*Read before the American Dental Society of Europe, April, 1912.

class of teeth to treat than those in which the foramen has been enlarged or which have been perforated into the alveolus either traumatically or by decay.

These cases are frequently supplemented by all the phenomena of supperative conditions either with or without fistulae.

Teeth of this class are usually considered hopeless and are condemned for extraction by most practitioners. I do not consider, however, that we have done our full duty towards our patients by such practice and we should neglect no means within our power to render such teeth healthy and useful members of the dental arch. Careful attention to my experience and method of procedure in such cases will, I am convinced, be useful to any one who is not familiar with it.

About thirty-six years ago the first case of perforation by decay came to my notice, and, as it was an important tooth (upper molar) I was very anxious to save it. It had a very large and deep cavity and the cementum was invaded by decay very low down below the margin of the alveolus in the center between the roots and a thorough removal of the decayed portion made an opening about four millimeters in diameter, which was quite sensitive and bleeding. I tried various methods of treatment in this case, such as putting gutta percha directly upon the opening; fitting a piece of gold and holding it in place with cement; a sheet of asbestos, etc., etc., but none of these were comfortable, or even tolerable. I did not want to extract the tooth and I finally thought of the comparative ease with which lead bullets become encysted and tolerated in the soft tissues and I hammered out a shot making a sheet of rather thick yet pliable lead and burnished this over my perforation. This was held firmly in place with an instrument while cement was placed upon it and within the cavity and allowed to harden.

I filled the tooth temporarily and waited developments before proceeding further. There was soreness for a few days, but much less than was experienced after any of the previous treatments, and this gradually disappeared, much to my own and the patient's gratification.

This tooth was finally restored with a filling and had given no sign of trouble nearly two years later, when I lost sight of the patient.

It may be noticed that in this case there was not, nor had there been, the slightest suppuration. It is needless to say that I have used this method ever since in the treatment of all similar perforations and almost all abscessed teeth, especially where the foramen is enlarged, either by disease or by me in the treatment of the abscess.

Some 18 years ago a gentleman presented himself with a lower molar sore and suppurating, which had been given up and condemned for extraction by another dentist. Examination showed enlarged foramen of distal root together with perforation by decay into periosteum between the roots. There was an abscess with fistula from the former and considerable suppuration from the latter. A careful measurement was taken of the exact length of the root by passing a "Donaldson" hook through the foramen and drawing it back till it caught on the end; the canal was then enlarged down to within less than a millimeter of the foramen, leaving a shoulder to prevent forcing the lead point through the opening. My lead point was carefully fitted always by exact measurement as to length; carbolic acid driven through the abscess until it appeared on the gum at the fistula, and the lead point cut off and driven down with a mallet as solidly as possible. The perforation between the roots was next treated with $H_2 O_2$ and the sheet lead burnished over it and fixed exactly as in case No. 1 and the tooth filled. No further suppuration ensued and the tooth was cured and entirely comfortable and has remained so ever since.

Another case treated by me in 1908, was a lateral incisor which had caused constantly recurring suffering during more than two years, notwithstanding many ineffectual attempts at treatment by ordinary methods. I opened the canal as usual and fitted a lead point, which I thought was surely long enough to reach the end of the canal, and as there was some sensitiveness and roughness to the use of the Donaldson hook in getting the first measurements of the length of the root, I feared even to have gone beyond the end. As there was little or no amelioration this radiograph was taken which showed me the exact conditions: A careful calculation of measurement was made and the canal opened fearlessly to the end. A new lead point was fitted, the abscess treated as elsewhere described, and the lead point driven home, since which time there has been no further trouble.

The root should be opened and enlarged with the Donaldson broach, if necessary, until the Donaldson hook will pass through the end. By drawing this latter instrument back until it catches on the end of the root an exact measurement is obtained, which is marked by a bit of rubber-dam slipped over the shaft of the hook beforehand. The canal is next enlarged by successive use of the different sizes of the Gates-Glidden drills as required.

A lead point is next fitted to the canal and marked with a notch. The abscess is then treated as you wish, and when ready to drive the lead point in solidly a groove is cut near the end and the point inserted and twisted off leaving the point at the end of the canal. This is then driven in by an instrument. A perforation is sometimes made in searching for the canal. Such a perforation in any root or in any tooth in the mouth is almost fatal; and the lead treatment I have described is the only one which gives almost sure hope of saving the tooth.

All traumatic lesions of this character are treated in the same manner with either lead points driven into the perforations or sheet lead burnished over them. I may remark here that in all the 36 years in which I have used lead for enlarged foramina and perforations I have never had but two cases where it was necessary to extract the tooth because of failure, that is, so far as I have any subsequent knowledge of the cases treated and the end of the root showed absorption in both. What physiological phenomena take place in these cases to determine and maintain a healthy condition it is exceedingly difficult to determine and could perhaps only be determined by the subsequent extraction of such teeth and submitting them to examination.

It seems likely that either these become encysted in the tissues, as in the case of lead balls, or else the exactness of the treatment and the mathematical certainty of having closed the openings with a pliable and non-irritating material produce the conditions which nature demands for the return to healthy and aseptic conditions.

My conclusions therefore, are that lead is more easily tolerated in contact with the periosteum or other sensitive tissues than any other material. That it is by far the best material to use because of the absolute certainty of exactly filling the enlarged canal to the end and no more, a thing impossible to accomplish in any other manner. This fact makes the saving of perforated teeth comparatively easy and facilitates the treatment of alveolar abscesses of whatever nature by permitting a free opening of the foramen for such treatment without fear of subsequent trouble after it is closed.—Abstract. of article in *The Dental Review*, October, 1912.

